# Identifying Genetic Architecture of Carcass and Meat Quality Traits in a Ningxiang Indigenous Pig Population

**DOI:** 10.3390/genes14071308

**Published:** 2023-06-21

**Authors:** Shishu Yin, Gang Song, Ning Gao, Hu Gao, Qinghua Zeng, Peng Lu, Qin Zhang, Kang Xu, Jun He

**Affiliations:** 1 College of Animal Science and Technology, Hunan Agricultural University, Changsha 410128, China; yinshishu2019@126.com (S.Y.); songgang19971109@163.com (G.S.); gaon@hunau.edu.cn (N.G.); gaohu_20190008@163.com (H.G.); chuweixiang168168@163.com (Q.Z.); qzhang@sdau.edu.cn (Q.Z.); 2 Laboratory of Animal Nutrition Physiology and Metabolism, The Chinese Academy of Sciences, The Institute of Subtropical Agriculture, Changsha 410128, China; 3 Center of Ningxiang Animal Husbandry and Fishery Affairs, Ningxiang 410625, China; sherryy2023@foxmail.com; 4 College of Animal Science and Technology, China Agricultural University, Beijing 100091, China

**Keywords:** genome-wide association study, carcass length, meat color, genetic parameter

## Abstract

Ningxiang pig is a breed renowned for its exceptional meat quality, but it possesses suboptimal carcass traits. To elucidate the genetic architecture of meat quality and carcass traits in Ningxiang pigs, we assessed heritability and executed a genome-wide association study (GWAS) concerning carcass length, backfat thickness, meat color parameters (L.LD, a.LD, b.LD), and pH at two postmortem intervals (45 min and 24 h) within a Ningxiang pig population. Heritability estimates ranged from moderate to high (0.30~0.80) for carcass traits and from low to high (0.11~0.48) for meat quality traits. We identified 21 significant SNPs, the majority of which were situated within previously documented QTL regions. Furthermore, the *GRM4* gene emerged as a pleiotropic gene that correlated with carcass length and backfat thickness. The *ADGRF1*, *FKBP5*, and *PRIM2* genes were associated with carcass length, while the *NIPBL* gene was linked to backfat thickness. These genes hold the potential for use in selective breeding programs targeting carcass traits in Ningxiang pigs.

## 1. Introduction

Carcass and meat quality traits are of paramount economic significance in the livestock industry. Carcass traits encompass backfat thickness (BFT), carcass length (CL), and other traits. Generally speaking, elevated body size in length and height is associated with heightened meat production. Compared to imported commercial breeds, most indigenous Chinese breeds exhibit smaller body sizes and lower meat production [1]. However, Chinese indigenous pig breeds possess superior meat quality and fat deposition, outperforming imported or crossbred pigs [2,3]. Notably, meat color and intramuscular fat deposition directly influence consumer perception and exhibit moderate-to-high heritability [4,5]. Previous studies have reported that “acid meat”, PSE (pale, soft, and exudative), and DFD (dark, firm, and dry) meat are seldom observed in indigenous pigs [6,7,8]. Meat color, tenderness, and water loss rate undergo the most significant changes, with the breed and pre- and postslaughter management being the primary factors contributing to PSE and DFD in pork [9]. Research indicates that the pH, drip loss, and meat color (lightness, redness, yellowness) of indigenous pigs surpass those of commercial pigs. Genetically, a few major genes have been identified as being associated with inferior meat quality, such as the *HAL^n^* gene (Halothane, or RYRI gene) and the *RN* (Renderment napole) gene, which profoundly impact PSE meat and acid meat [10,11].

The genetic architecture characterizes the phenotype alterations resulting from genetic variation, with specific research areas encompassing the number of variations impacting traits, population occurrence frequency, the genetic effect’s scope, and relationships with other genes (additive and interactive effects) or environmental factors [12,13]. Exploring the genetic architecture of complex quantitative traits aids in the detection of novel single-nucleotide polymorphisms (SNPs) or genes associated with these traits. A genome-wide association study (GWAS) represents a prevalent approach for comprehending the genetic architecture of quantitative traits and for discovering new genes. Prior research has identified numerous candidate genes associated with economic traits, such as carcass traits [14,15], meat quality traits [5,16], and reproductive traits [17,18]. While Ningxiang pig is renowned for its meat quality and disease resistance, it exhibits a suboptimal growth rate and lean meat percentage. Deciphering the genetic architecture of these economic traits could facilitate the genetic enhancement of Ningxiang pigs’ shortcomings while preserving their advantages through marker-assisted selection, ultimately benefiting the Ningxiang pig industry. In this study, we performed a GWAS on carcass and meat quality traits within a Ningxiang pig population, identifying several candidate genes related to these traits, which hold the potential for implementation in Ningxiang pig breeding programs.

## 2. Materials and Methods

### 2.1. Phenotypes and Genotyping

Phenotypic data were collected for Ningxiang pigs (*n* = 508, including 21 females and 487 males) that were slaughtered at a predetermined age (180 ± 5 days) from the Ningxiang Chu Weixiang Slaughterhouse and Meat Processing, LLC (Ningxiang, Hunan Province, China). Carcass traits included left half carcass weight (LW), carcass oblique length (COL), carcass length (CL), and backfat thickness (BFT), measured by the national technical regulation for testing of carcass traits in lean-type pig (NY/T 825-2004). Meat quality traits, such as three meat color parameters (L.LD, a.LD, b.LD) of longissimus dorsi (i.e., lightness, redness, and yellowness) at 45 min after slaughter and pH of longissimus dorsi at two postmortem time points (45 min and 24 h), were assessed following the national technical regulation for determination of pork quality (NY/T 821-2019). Detailed measurement results and methods for carcass and meat quality traits are presented in Table 1 and Table S1.

Genomic DNA was extracted from muscle tissue using standard phenol chloroform method, and the DNA was dissolved in TE buffer. The Nanodrop 2000 spectrophotometer was used to measure the concentration and purity of DNA samples. The samples with A_260/280_ ratio between 1.7~2.0 were genotyped using the GeneSeek Genomic Profiling (GGP) version 2 Porcine 50K SNP chip (Neogen Corporation, Lincoln, NE, USA), which comprises 50,697 SNP loci.

### 2.2. Genotype Imputation and Quality Control

To reduce the missing genotype rate, we employed Beagle5.4 software [19] to impute the missing genotypes. Subsequently, quality control was conducted using PLINK v1.9 [20] with the following criterion: (1) SNP call rate ≥ 90%; (2) minor allele frequency (MAF) ≥ 1%; (3) Hardy–Weinberg equilibrium (HWE) testing *p*-value ≤ 10^−6^; (4) on autosomes with known positions. After quality control, 537 and 14,812 SNPs were removed due to HWE and MAF thresholds, respectively. Additionally, 4197 SNPs located on the sex chromosome or with unknown chromosome positions were excluded. Ultimately, 31,106 SNPs distributed across 18 autosomes remained for association analysis (Figure S1). More details about the SNP distribution are presented in Table S2.

### 2.3. Statistical Method

#### 2.3.1. Estimation of Genetic Parameters

The heritabilities and genetic correlations for the studied traits were estimated using the multiple-traits model of the HIBLUP software [21]. The model follows [21]:

(1) $$y=Xb+Rr+\sum_{i=1}^{k}Z_{i}u_{i}+e;r\sim N\left(0,I\sigma_{r}^{2}\right);u_{i}\sim N\left(0,K_{i}\sigma_{i}^{2}\right);e\sim N(0,{I\sigma }_{e}^{2})$$

where ***y*** is the vector of phenotypic data; ***X*** and ***R*** are the design matrix for fixed effects (including covariates) and environmental random effects, respectively; ***b*** and ***r*** are the vector of corresponding and estimated effects. ***Z****_i_* is the design matrix for the *i*-th genetic random effect and ***u****_i_* is the vector of its responding genetic effects. ***K****_i_* is the additive genetic relationship matrix, ***I*** is an identity matrix, and ***e*** is the vector of residual errors. The heritability (h^2^), genetic correlation (*r_A_*), and phenotypic correlation (*r_P_*) are calculated by $\frac{\sigma_{a}^{2}}{\sigma_{a}^{2}+\sigma_{e}^{2}}$, $\frac{Cov(a_{1},a_{2})}{\sqrt{\sigma_{a1}^{2}\sigma_{a2}^{2}}}$, and $\frac{Cov(p_{1},p_{2})}{\sqrt{\sigma_{p1}^{2}\sigma_{p2}^{2}}}$, where $\sigma_{a}^{2}$ and $\sigma_{e}^{2}$ are the additive genetic variance and residual variance, respectively. *Cov*(***a***_1_, ***a***_2_) is the additive effect covariance between ***a***_1_ and ***a***_2_ traits, and *Cov*(*p*_1_, *p*_2_) is the phenotypic covariance between *p*_1_ and *p*_2_ traits.

#### 2.3.2. Principal Component Analysis

To avoid hidden population stratification causing false positives in GWAS, we used imputed genotypes to perform principal component analysis (PCA) with PLINK v1.9 (command: --pca). The results depicted in Figure S2 suggest that this population may have population stratification, and PCs need to be added for correction.

#### 2.3.3. Genome-Wide Association Study

GWAS was conducted using the rMVP package [22]. Sex was treated as fixed effects, and CW and five PCs were treated as covariates. We assessed the association between phenotypes and each SNP across the genome under the following linear mixed model (MLM) [23,24]:

(2) $$y=Xb+Za+u+e;u\sim N(0,G\sigma_{a}^{2});e\sim N(0,I\sigma_{e}^{2})$$

where ***y*** is a vector of phenotypic observations, ***b*** is a vector of fixed effects (included sex, CW, and 5 PCs), ***a*** is a vector of SNP effects; ***u*** is a vector of random polygenic effects with a covariance structure; ***e*** is a vector of residual errors. ***X***, ***Z*** are the design matrix of fixed and SNP effects, respectively. $\sigma_{a}^{2}$ and $\sigma_{e}^{2}$ are additive genetic and residual variances, respectively. ***I*** is an identity matrix, and ***G*** is the genomic relationship matrix calculated by the following [25]:

(3) $$G=\frac{ZDZ\prime }{\sum_{j=1}^{k}2p_{j}(1-p_{j})}$$

where ***Z*** is the matrix related to genotypes of each SNP (encoded 0, 1, 2 for AA, AB, and BB, respectively); ***D*** is a diagonal matrix of weights for SNP variance; *k* is the number of SNPs; *p_j_* is the minor allele frequency at *j*-th loci. The genome-wide and suggestive significant thresholds were 0.05/N_SNP_ and 1/N_SNP_, respectively. The proportion of variance explained (PVE) by a SNP was defined as follows [26]:

(4) $$PVE=\frac{2{\hat{\alpha }}^{2}MAF(1-MAF)}{2{\hat{\alpha }}^{2}MAF(1-MAF)+{\left(se(\hat{\alpha })\right)}^{2}2NMAF(1-MAF)}$$

where $\hat{\alpha }$ is the effect size for SNP marker, *MAF* is the minor allele frequency for SNP marker, $se(\hat{\alpha })$ is standard error of effect size for SNP marker, and *N* is the sample size.

### 2.4. Linkage Disequilibrium Analysis

To detect the linkage disequilibrium (LD) between significant SNPs, SNPs centering on each significant SNP was utilized to conduct LD analysis using the LDblockShow software (v 1.40) [27].

### 2.5. Candidate Genes Related to Significant SNPs

To identify candidate genes near the significant SNPs, we examined the annotated genes within a 500 kb radius round each SNP in the Sus scrofa 11.1 genome, using the biomaRt package (https://bioconductor.org/packages/3.15/bioc/html/biomaRt.html accessed on 5 July 2022). To annotate significant SNPs located in previously mapped QTLs in pigs, all QTL data in pigs were downloaded from the animal QTLdatabase. (https://www.animalgenome.org/cgi-bin/QTLdb/SS/download?file=gbpSS_11.1 accessed on 5 July 2022). Kyoto Encyclopedia of Genes and Genomes (KEGG) and Gene Ontology (GO) analyses were employed to identify related pathways. KEGG and GO analyses were performed using KOBAS [28] and AmiGO2 (http://amigo.geneontology.org/amigo/ accessed on 5 July 2022). To obtain more comprehensive gene enrichment results, we used the Homo Sapiens database for GO and KEGG pathway enrichment. The Benjamini–Hochberg procedure was used to correct the significance of the enriched terms, with *p*-adj < 0.05 as the significant threshold.

## 3. Results

### 3.1. Descriptive Statistics of Phenotypes

Descriptive statistics of carcass and meat quality traits of Ningxiang pigs are presented in Table 1. All phenotypic data conformed to the Gaussian distribution before GWAS (Figure S3). Substantial phenotypic variations were observed, with the coefficient of variation (CV) ranging from 4.74% to 41.75% for the eight traits.

### 3.2. Estimates of Genetic Parameters

The estimates of the heritabilities of these traits and the phenotypic and genetic correlations between them are shown in Table 2. In phenotype correlations, CL and COL were significantly negatively correlated with BFT (*r* = −0.12, *p* < −0.001; *r* = −0.16, *p* < −0.001), and except L.LD (lightness), carcass and meat color traits exhibited an extremely significant negative or positive correlation. L.LD only demonstrated a significant negative correlation with two pH traits, and positive correlations with a.LD and b.LD. BFT also exhibited a negative correlation with CL and COL in genetic correlations. CL showed a negative correlation with pH traits and a positive correlation with L.LD. In this study, the heritabilities (±SE) of carcass traits were moderate to high and ranged from 0.47 (±0.07) to 0.80 (±0.07), and meat quality traits demonstrated low-to-high heritability, ranging from 0.11 (±0.07) to 0.44 (±0.08).

### 3.3. GWAS Results and Gene Annotation

**Figure 1:**
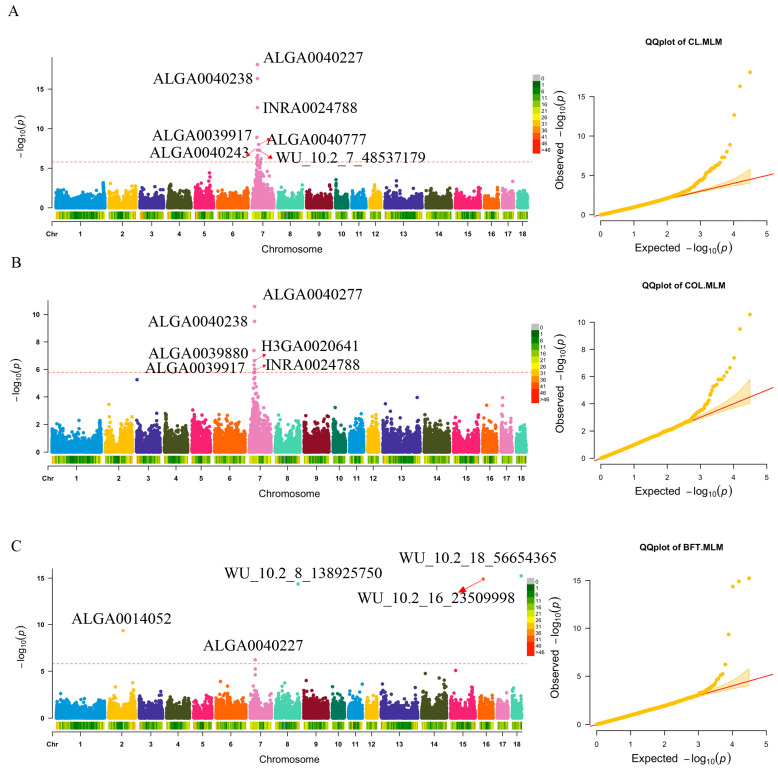
Manhattan and Q-Q plots for three carcass traits. (**A**–**C**) are CL, COL, and BFT traits, respectively. The red dashed line is the genome-wide threshold −log_10_(0.05/31,106). Because of the overlap of SNPs, some significant SNPs in CL trait are not marked. The λ represents genomic inflation factors. The red arrow indicates significant SNP loci.

**Figure 2:**
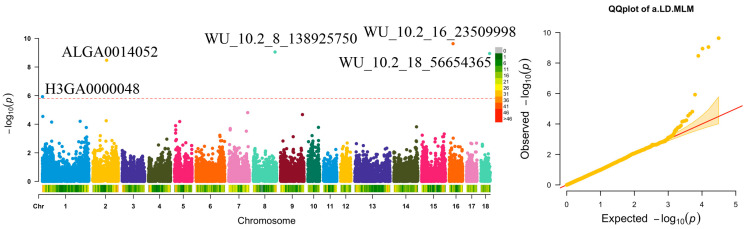
Manhattan and Q-Q plots s for meat trait (a.LD). The dashed red line is the genome-wide threshold −log_10_(0.05/31,106). The λ represents genomic inflation factors.

After quality control, 31,106 SNPs were available for subsequent GWAS. The average physical distance between two neighboring SNPs was approximately 71 kb and ranged from 55 kb (SSC7) to 82 kb (SSC1) (Table S2). Single-marker tests using MLM were performed to identify genetic markers associated with these traits at the genome-wide significant level (threshold = 0.05/31,106). The GWAS results are presented in Figure 1, Figure 2 and Figure S4, as well as Table 3 and Table S3. By adding five PCs as covariates, the Q-Q plots of *p*-values and the computed genomic inflation factors (λ) indicated no evidence of population stratification.

#### 3.3.1. Carcass Trait

For CL and COL, 15 and 6 genome-wide significant SNPs were identified on SSC7, respectively (Table 3a). ALGA0040227 was the most significant SNP for CL and COL traits, contributing 14.35% and 8.38% to the phenotypic variance. Among all the significant SNPs, eight loci were intergenic (located within *GRM4*, *MLIP*, *FKBP5*, *PRIM2*, *TINAG*, and *ZNF76*, respectively). Additionally, the most significant SNPs were intron variants; a few belonged to unknown variants (INRA0024788, WU_10.2_7_48537179, and WU_10.2_7_36255497). For BFT, there were five genome-wide significant SNPs identified and distributed on SSC2, SSC7, SSC8, SSC16, and SSC18, respectively (Figure 1C), WU_10.2_18_56654365 was the most significant SNP, contributing 12.66% to the phenotypic variance. Two SNPs (WU_10.2_18_56654365, WU_10.2_16_23509998) were located within the *HECW1* and *NIPBL* genes, respectively.

#### 3.3.2. Meat Quality Trait

Only the a.LD trait identified five genome-wide level significant SNPs, located on SSC1, SSC2, SSC8, SSC16, and SSC18, respectively. The most significant SNP was WU_10.2_16_23509998, located on SSC16 (Figure 2 and Table 3b), contributing 7.95% of the phenotypic variance. Four of these loci (WU_10.2_16_23509998, WU_10.2_8_138925750, WU_10.2_18_56654365, and ALGA0014052) were also associated with BFT. No significant SNPs were found for the other traits in this study (Figure S4).

### 3.4. LD Block Analysis

**Figure 3:**
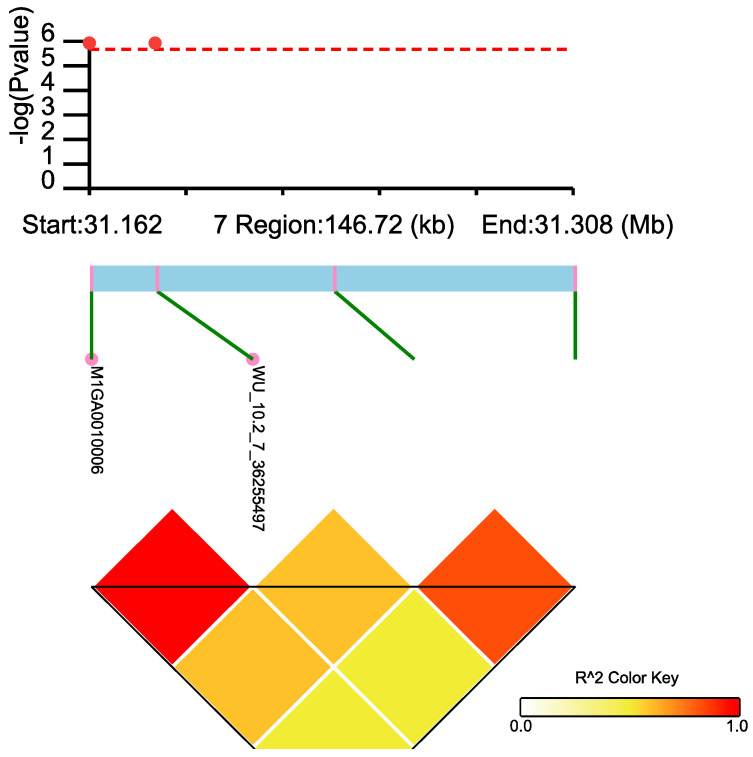
The LD block in the significantly associated region on SSC7. LD blocks are marked with triangles. Values in boxes are LD (*r*^2^) between SNP pairs. From 31161760 to 32150539, only two significant SNPs, M1Ga0010006 and WU_10.2_7_36255497, are within the LD block, and their LD value is 1. The dashed red line represents 0.05/31,106 threshold, and the red dot represents genome-wide level SNP.

Twelve LD blocks were identified in regions 26.50–50.28 Mb on SSC7, but only one block included two genome-wide significant SNPs and indicated strong LD (*R*^2^ = 1). LD block analysis revealed that the multiple significant SNPs on SSC7 associated with CL spanned 146.72 kb (*R*^2^ = 0.3) (Figure 3).

### 3.5. Functional Enrichment Results

**Figure 4:**
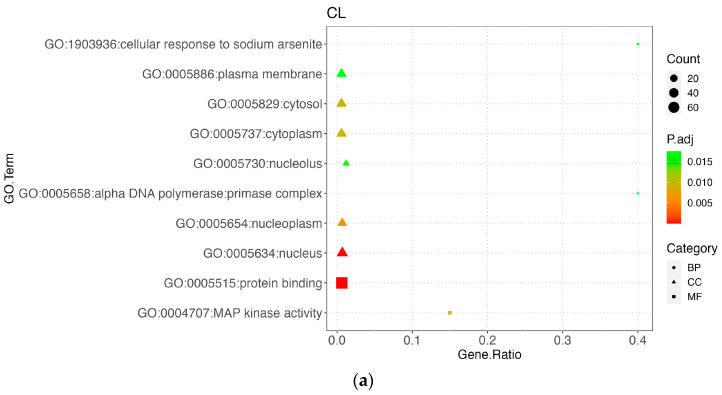

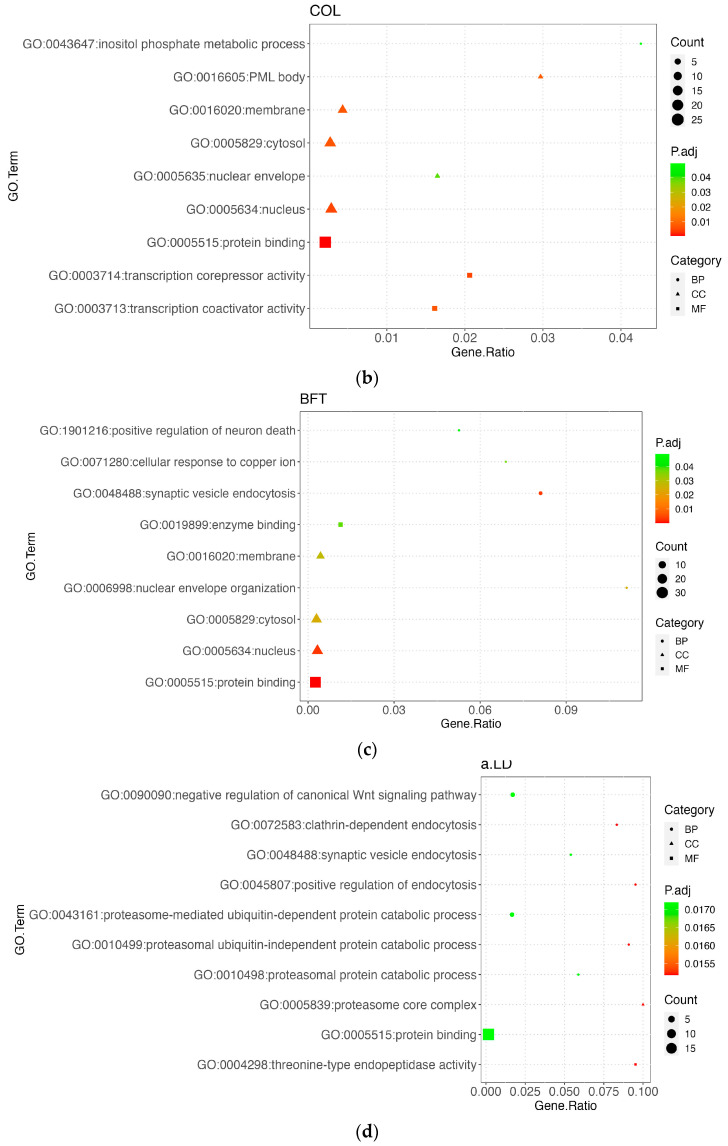
The GO enrichments of CL (**a**), COL (**b**), BFT (**c**) and a.LD (**d**) traits. Different shapes represent different categories: circle represents biological process (BP), triangle represents cellular component (CC), and square represents molecular function (MF).

To annotate the potential SNPs, candidate genes overlapping with the extended genomic regions were selected for GO term enrichment analysis. A total of 135 genes were identified in carcass traits (CL, COL, and BFT) and 32 genes in meat color a.LD (redness). However, only 10 SNPs were located within 8 genes. (Top 10 GO terms shown in Figure 4a–d, KEGG pathway shown in Table S4).

#### 3.5.1. Carcass Trait

A total of 112 genes overlapped with or were close to the significant SNP loci for the CL trait. Most of these genes were significantly enriched in GO terms of biological processes (BP), followed by cellular components (CC). There were three significant KEGG pathways: amyotropic lateral sclerosis (ALS), spliceosome, and cellular sensitivity.

For COL, there were 37 potential genes within these genomic regions. For BFT, there were 43 genes within 1Mb genomic regions, and these genes were significantly enriched in only one pathway (Glutamatergic synapse). Protein binding (GO:0005515) was the most enriched GO term among the three carcass traits (Figure 4a–c).

#### 3.5.2. Meat Quality Trait

Thirty-two genes were used for enrichment analysis for a.LD, and biological process (BP) was the most enriched category in the top 10 GO terms (Figure 4d). Proteasome and RNA transport were the only two significant KEGG pathways.

## 4. Discussion

In this study, the heritability of carcass traits ranged from 0.47 to 0.80, while meat quality traits ranged from 0.11 to 0.44. The genetic parameters obtained in this study for carcass and meat quality traits demonstrated congruence with previous studies [29,30,31]. Carcass and meat quality are the livestock industry’s most crucial economic target traits. The Ningxiang pig, celebrated for its superior meat quality and robust disease resistance, nonetheless manifests a suboptimal growth rate, a relatively short body length, and a lean meat percentage. Furthermore, backfat thickness (BFT), a significant component of carcass traits, substantially influences reproduction and meat production performances [14,32]. To maintain consistency with consumer demands, reducing backfat thickness and improving lean meat percentage and growth rate have become the goals of breeders [33]. Concurrently, keeping high-quality pork is also essential. A significant negative correlation was discerned between BFT and body length in phenotypic and genetic correlation results. Most Chinese indigenous pig breeds were shorter than the imported breeds but had thicker BFT [34]. Ningxiang pigs, a famous obese pig breed, have a BFT (41.61 mm) thicker than commercial breeds [15,35], and are comparable to Chinese indigenous obese breeds [36]. In this study, the average carcass length (81.35 cm) was shorter, and BFT (41.61 mm) was thicker than commercial breeds [4]. Concerning meat quality traits, meat color mainly described three parameters, namely L, a, and b, denoting lightness, redness, and yellowness, respectively. Redness is associated with myoglobin content, with elevated myoglobin presenting increased redness [37]. A previous study found the meat color of Ningxiang pigs comparable to that of Chinese Sutai pigs [38]. Compared to Duroc pigs, Ningxiang pigs exhibited higher redness, yellowness, and lightness [35]. In phenotypic correlation, most carcass traits and pH traits showed a significant negative correlation with meat color traits, while genetic correlation differed. pH_45min_ and pH_24h_ were negatively genetically correlated with redness, and yellowness, respectively. The study revealed that low acidity could affect meat color, structure, and tenderness [39], consistent with our team’s previous report [40]. Additionally, there was a negative correlation between lightness and BFT. Yuan et al. reported that polymorphisms in the *DGAT1* gene affected meat color, known for its role in fat deposition [41].

In this study, we performed a GWAS in a Ningxiang population to explore the genetic architecture of carcass and meat quality traits. We identified 21 genome-wide significant SNPs and several candidate genes for carcass traits (CL, COL, BFT) and one meat quality trait (a.LD). We identified some novel SNPs and genes potentially associated with these traits, which had no research previously. Therefore, it is essential to conduct GWAS in different pig breeds to identify more genes underlying the complex traits, which would benefit Ningxiang pig breeding programs. Previous studies concluded that some SNP-containing annotated genes were highly associated with carcass and meat quality traits. Notably, we found that some SNPs exhibited pleiotropy in multiple traits. Watanabe et al. [42] indicated that numerous pleiotropy loci, SNPs, or genes existed between traits with solid correlations, especially within the same domain. For example, CL was highly correlated with COL in phenotype and genetics; we identified six SNPs for two traits, and ALGA0040227 was also an important site for BFT. A total of 113 reported QTLs were within this genomic region, with 3 associated with carcass length [43,44], 16 QTLs related to backfat thickness [45,46], and 3 QTLs associated with meat color [16,47]. ALGA0040227 was closest to the *GRM4* (Glutamate metabotropic receptor 4) gene. Metabolic glutamate (mGlu) receptors are a family of G protein-coupled receptors that regulate cell physiology throughout the nervous system [48]. GRM4 belongs to a subtype of the Metabotropic glutamate receptor family, and is mainly involved in maintaining the stability of the internal environment of central nervous system cells [49]. This gene plays an important role in various cancers, such as melanoma [50], breast cancer [51], and osteosarcoma [52]. Osteosarcoma is the most common primary malignant tumor of bone, which occurs in the long bones of the limbs and tends to occur at the peak of adolescent growth [53]. Maya et al. [54] found that GRM4 played an important role in driving osteosarcoma by regulating the noncellular autonomous mechanism of IL-23, which opened up a new direction for treatment. Additionally, Wang et al. [14] indicated that the *GRM4* gene may play an essential role in adipogenesis by activating MAPK activity.

In this study, all significant SNPs were located within or near several genes (*HMGA1*, *MLIP*, *UNC5CL*, *ADGRF1*, *FKBP5*, *PRIM2*, *TINAG*, *TMC3*, *SNCA*, *SRSF3*, *ZNF76*, and *ERMARD*). Some of these genes have been reported to be associated with interesting phenotypes. For example, The HMGA1 (high-mobility group AT-hook 1) gene is a nonhistone chromatin structural protein characterized by the absence of transcriptional activity, and belongs to the high-mobility family A, which comprises three members: HMGA1, HMGA2, and HMGA3. This gene plays a vital role in osteoblast commitment and mediates the function of NFIX by transcriptionally activating canonical Wnt signaling [55]. Moreover, the *HMGA1* gene is a vital regulator of the insulin receptor (*INSR*) gene [56]. This gene has been reported to be related to many traits. For example, Ding et al. [15], Wang et al. [14], and Kim et al. [57] reported that *HMGA1* was associated with fat deposition traits in pigs. Additionally, this gene has been reported to be associated with obesity [58], diabetes [59], and metabolic syndrome [56] in humans. Gong et al. [60] and Liu et al. [61] reported that this gene was associated with growth traits (e.g., cannon circumference and body length) and body size in pigs. Otto et al. [62] identified that the *HMGA1* gene affected the measurement of meat color. In this study, BFT and carcass length traits also exhibited strong phenotypic and genetic correlations. The *ADGRF1* gene, also known as the *GPR110* gene, is a member of the adhesion GPCR family, and functions as a receptor of N-docosahexaenoyl ethanolamine [63]. Hidaka et al. [64] suggested that synaptamide/GPR110 signaling negatively regulates osteoclastogenesis. This gene has also been reported to be associated with carcass length in pigs [65]. *PRIM2* (DNA primase subunit 2, also named *PRIM2A*) encodes 58 kDa protein containing a 4Fe-4S cofactor that forms a heterodimeric DNA primase with PRIM1, a small subunit of DNA primase [66]. Wang et al. [67] identified the *PRIM2* gene as associated with body length. The *FKBP5* gene (FKBP prolyl isomerase 5, all named *AIG61*, *FKBP54*) encodes the FKBP5 protein, an immunoaffinity protein with multiple biological functions. Lu et al. [68] found that the *FKBP5* gene is involved in NF-kB and Akt signaling pathways, which regulate and control osteoclasts differentiation and development. They also pointed out that the *FKBP5*^V55L^ mutation is related to osteoclastogenesis and function, which affects the development of Paget’s disease. This gene is a potential candidate for skeletal muscle development. The *MLIP* (Muscular A-type Lamin interacting protein, also called *MMCKR* or *CIP*) gene encodes alternatively spliced variants (23–57 kDa) with several novel structural motifs not found in other proteins, and is highly expressed in heart, skeletal, and smooth muscle [69]. Huang et al. [70] identified it as a candidate gene for the forming of exterior traits (facial wrinkles) in Chinese Erhualian pigs.

Furthermore, few studies have investigated these genes in livestock or their association with interesting phenotypes, such as *TMC3* (transmembrane channel-like 3), *SNCA* (α-synuclein), *TINAG* (Tubulointerstitial nephritis antigen, also named *TIN-AG*) and *ZNF76* (Zinc finger protein 76) genes. The *TINag* gene encodes an extracellular matrix protein, TINag, which is expressed in tubular basement membranes [71]. Most studies on this gene have focused on disease. For instance, Tong et al. [71] identified a mutation in *TINAG* as a prognostic biology marker for pectus excavatum (PE). Jakowlev et al. [1] suggested that *TINAG* might be a potential susceptibility gene for hand osteoarthritis. The *UNC5CL* gene (all called *MUXA*, *ZUD*) is a member of the UNC5 family, and has a unique death and ZU5 domain in its molecular structure. It is also involved in immunity and inflammation [72]. This gene has been extensively implicated in mucosal diseases [73,74].

For the BFT trait, we identified five candidate genes (*HECW1*, *NIPBL*, *SNCA*, *TMEM174*, *GRM4*), of which four were also found in a.LD, including *HECW1*, *NIPBL*, *SNCA*, *TMEM174* genes. Additionally, two significant SNPs were located within *NIPBL* (nipped-B-like protein cohesin loading factor) and *HECW1* (HECT, C2, and WW domain-containing E3 ubiquitin protein ligase 1, also called *NEDL1*), respectively. The *NIPBL* encodes the homolog of Nipped-B-like protein and colon tumor susceptibility 2-type sister chromatid cohesion proteins, facilitating enhancer–promoter interaction of remote enhancers. It is highly expressed in the lung, spleen, and subcutaneous adipose tissue. This study discovered that the *NIPBL* gene was enriched in embryo development, such as embryonic viscerocranium morphogenesis (GO:0048703) and embryonic digestive tract morphogenesis (GO:0048557). Alonso-Gil et al. [75] reported that low-level NIPBL seriously affects genome folding. In farm animals, this gene has been reported to be associated with limb development in Qinchuan cattle [76], milk traits in Chinese dairy cattle [77], and adipogenesis in Duroc pigs [78]. *HECW1* was highly expressed in the kidney and ovary and is one of nine HECT ubiquitin-like ligase NEDD4 family members. No studies have shown that this gene is related to traits of interest. The other two genes, *SNCA* and *TMEM174* (Transmembrane protein 174), are also unrelated to fat deposition or meat color formation.

Meat color is a significant factor affecting consumer preferences. Redness, yellowness, and lightness serve as primary indicators of meat color. Factors influencing meat color include pigment sources such as myoglobin, hemoglobin, cytochrome C, and muscle structure [79]. In this study, we identified significant loci for only one meat color trait (a.LD), with candidate genes associated with iron ion transport, mitochondrial cytochrome c oxidase assembly, and negative regulation of myoblast differentiation. However, the obtained genes have no studies about meat color.

We searched the pig QTL database based on SNP and QTL locations to assess whether this study’s SNPs associated with carcass and meat quality traits replicated any previously known QTLs. We identified 21 SNPs associated with carcass and meat quality traits within genomic regions. The top 10 traits with the highest enrichment are shown in Table S5, with average daily gain exhibiting the highest enrichment among all traits. Reported QTLs associated with carcass traits were found in genomic regions for CL and COL. Average backfat thickness, fat cut percentage, and intramuscular fat content were related to fatness and meat quality for BFT. Some QTLs for meat color traits (L, a, and b) were also identified in the a.LD genomic region.

## 5. Conclusions

Through a genome-wide association study on carcass and meat quality traits in a Ningxiang pig population, we detected 21 SNPs associated with the traits of interest and identified several candidate genes related to these SNPs. *GRM4* emerged as a potential pleiotropic gene associated with carcass length and BFT. *HMGA1*, *ADGRF1*, *FKBP5*, and *PRIM2* genes were identified as associated with carcass length, while the *NIPBL* gene was associated with BFT. These findings contribute to a better understanding of the genetic architecture of carcass and meat quality traits in Ningxiang pigs and hold the potential for application in inbreeding programs.

## Figures and Tables

**Table 1:** Summary statistics for carcass and meat quality traits in Ningxiang pigs.

Trait	*n*	Max.	Min.	Mean ± SD	C.V.
CL (cm)	508	96.40	68.50	81.35 ± 4.69	5.77
COL (cm)	508	86.50	34.10	66.11 ± 6.16	9.32
BFT (mm)	485	71.06	16.17	41.61 ± 8.28	19.90
L.LD	508	58.73	34.80	44.73 ± 3.84	8.58
a.LD	508	16.17	1.34	6.53 ± 2.61	39.97
b.LD	508	10.53	0.14	4.00 ± 1.67	41.75
pH_45min_	508	6.96	5.46	6.28 ± 0.31	4.94
pH_24h_	508	6.87	5.46	5.91 ± 0.28	4.74

**Table 2:** Heritability estimates and genetic and phenotypic correlation coefficients among studied traits.

Trait	CL	COL	BFT	pH_45min_	pH_24h_	L.LD	a.LD	b.LD
CL	0.80 (0.06)	0.87	−0.53	−0.22	−0.27	0.25	−0.05	0.14
COL	0.82 ***	0.47 (0.07)	−0.53	0.08	−0.47	0.07	−0.41	0.50
BFT	−0.12 **	−0.16 ***	0.48 (0.08)	0.07	−0.08	−0.32	0.07	−0.32
pH_45min_	0.01	0.02	−0.04	0.14 (0.11)	0.10	0.27	−0.39	0.41
pH_24h_	−0.05	−0.09	0.12	0.37 ***	0.30 (0.09)	0.45	0.42	−0.37
L.LD	0.06	−0.03	−0.07	−0.24 ***	−0.2 ***	0.11 (0.07)	0.38	−0.08
a.LD	−0.11 *	−0.28 ***	0.18 ***	−0.07	−0.04	0.31 ***	0.44 (0.08)	−0.23
b.LD	0.27 ***	0.32 ***	−0.18 ***	0.09	−0.24 ***	0.24 ***	0.03	0.19 (0.09)

Lower triangle numbers are phenotypic correlation, upper are genetic correlation, and the diagonal line represents heritability (±SE) of each trait. “***”, “**”, and “*” indicate *p* < 0.001, *p* < 0.01, and *p* < 0.05, respectively.

**Table 3:** (**a**) The genome-level significant SNPs and possible candidate genes for carcass traits. (**b**) The genome-level significant SNPs and possible candidate genes for meat quality traits.

(a)									
Trait	SNP (Rsid)	CHR	POS (bp)	Consequence	MAF	PVE (%)	*P-adj*	Nearest Gene	DIS (bp)
CL	ALGA0040227 (rs80983858)	7	30,176,520	Downstream gene variant	0.39	14.35	8.05 × 10^−19^	*GRM4*	2785
	ALGA0040238 (rs80815545)	7	30,197,014	Intron variant	0.36	12.98	4.72 × 10^−11^	*GRM4*	Within
	INRA0024788 (–––)	7	30,31,7219	–––	0.36	10.08	2.16 × 10^−13^	*HMGA1*	3191
	ALGA0039917 (rs81397589)	7	26,737,102	Intron variant	0.19	7.02	1.25 × 10^−9^	*MLIP*	Within
	ALGA0040777 (rs80845178)	7	36,323,988	Intergenic variant	0.44	6.28	9.85 × 10^−9^	*UNC5CL*	8213
	ALGA0040243 (rs80942143)	7	30,213,771	Intron variant	0.25	5.69	4.97 × 10^−8^	*GRM4*	Within
	WU_10.2_7_48537179 (–––)	7	41,877,149	–––	0.42	5.66	5.39 × 10^−8^	*ADGRF1*	23,492
	ASGA0032589 (rs80869188)	7	31,450,019	Intron variant	0.32	5.13	2.36 × 10^−7^	*FKBP5*	Within
	H3GA0020641 (rs80975871)	7	28,521,421	Intron variant	0.11	4.92	4.17 × 10^−7^	*PRIM2*	Within
	ALGA0039880 (rs80928470)	7	26,501,975	Intron variant	0.11	4.86	5.04 × 10^−7^	*TINAG*	Within
	ALGA0041948 (rs80997002)	7	50,283,279	Intergenic variant	0.47	4.77	6.30 × 10^−7^	*TMC3*	99,190
	ALGA0040370 (rs81397836)	7	32,328,188	Intergenic variant	0.48	4.60	1.02 × 10^−6^	*SRSF3*	29,608
	M1GA0010006 (rs80946246)	7	31,161,760	Intron variant	0.31	4.55	1.16 × 10^−6^	*ZNF76*	Within
	WU_10.2_7_36255497 (–––)	7	31,181,718	––––	0.31	4.55	1.16 × 10^−6^	*ZNF76*	Within
	MARC0060950 (rs80924014)	7	46,569,153	Upstream gene variant	0.16	4.44	1.58 × 10^−6^	*TMEM14A*	51,421
COL	ALGA0040227 (rs80983858)	7	30,176,520	Downstream gene variant	0.39	8.38	2.67 × 10^−25^	*GRM4*	2785
	ALGA0040238 (rs80815545)	7	30,197,014	Intron variant	0.36	7.51	3.17 × 10^−10^	*GRM4*	Within
	ALGA0039880 (rs80928470)	7	26,501,975	Intron variant	0.11	5.75	4.19 × 10^−7^	*TINAG*	Within
	H3GA0020641 (rs80975871)	7	28,521,421	Intron variant	0.11	5.14	2.30 × 10^−7^	*PRIM2*	Within
	ALGA0039917 (rs81397589)	7	26,737,102	Intron variant	0.19	4.88	4.74 × 10^−7^	*MLIP*	Within
	INRA0024788	7	30,317,219	–––	0.36	4.63	9.33 × 10^−7^	*HMGA1*	3191
BFT	WU_10.2_18_56654365 (–––)	18	51,759,775	–––	0.12	12.66	5.80 × 10^−16^	*HECW1*	Within
	WU_10.2_16_23509998 (–––)	16	22,361,911	–––	0.12	12.38	1.27 × 10^−15^	*NIPBL*	Within
	WU_10.2_8_138925750 (–––)	8	129,537,879	–––	0.12	11.94	4.37 × 10^−15^	*SNCA*	266,751
	ALGA0014052 (rs81360052)	2	82,412,427	Intron variant, noncoding transcript variant	0.14	7.72	4.52 × 10^−10^	*TMEM174*	75,272
	ALGA0040227 (rs80983858)	7	30,176,520	Downstream gene variant	0.39	5.01	6.14 × 10^−7^	*GRM4*	2785
**(b)**									
**Trait**	**SNP**	**CHR**	**POS (bp)**	**Consequence**	**MAF**	**PVE (%)**	***P-adj***	**Nearest Gene**	**DIS (bp)**
a.LD	WU_10.2_16_23509998 (–––)	16	22,361,911	–––	0.12	7.94	2.34 × 10^−10^	*NIPBL*	Within
	WU_10.2_8_138925750 (–––)	8	129,537,879	–––	0.12	7.44	8.95 × 10^−10^	*SNCA*	266,751
	WU_10.2_18_56654365 (–––)	18	51,759,775	–––	0.12	7.36	1.14 × 10^−9^	*HECW1*	Within
	ALGA0014052 (rs81360052)	2	82,412,427	Intron variant, noncoding transcript variant	0.14	6.95	3.38 × 10^−9^	*TMEM174*	75,272
	H3GA0000048 (rs80803041)	1	493,510	Intergenic variant	0.01	4.75	1.19 × 10^−6^	*ERMARD*	19,168

## Data Availability

The data presented in this study are available on request from the corresponding author.

## Supplementary Materials

The following are available online at: https://www.mdpi.com/article/10.3390/genes14071308/s1, Figure S1: SNP density after quality control; Figure S2: Principal component analysis of 508 animals; Figure S3: Phenotypic distribution of eight traits, Sd represents standard deviation; Figure S4: Manhattan and Q-Q plots for 4 traits (b.LD, L.LD, pH_45min_, pH_24h_). The red line is the genome-wide threshold (0.05/31,106). The −log10(*p*-value) of each SNP (*y*-axis) across the chromosomes (*x*-axis), along with the corresponding Q-Q plots. The λ represents genomic inflation factors. Table S1: Abbreviation and measurement method description in this study; Table S2: Distribution of SNPs before, and after quality control and the average distance between adjacent SNPs on each chromosome; Table S3: The genome-level significant and possible candidate genes for carcass and meat quality traits; Table S4: Enrichment of KEGG pathway in Homo Sapiens dataset; Table S5: Top 10 traits with the highest enrichment QTLs number.
