# Supplementary material for: Identifying Genetic Architecture of Carcass and Meat Quality Traits in a Ningxiang Indigenous Pig Population
Source: Genes (Basel). 2023 Jun 21;14(7):1308. doi: 10.3390/genes14071308 (PMC10378861; doi:10.3390/genes14071308)
Supplement: Supplementary file 1 [file genes-14-01308-s001.zip › genes-2419942 - Supplementary/genes-2419942 - Supplementary Figure.docx]

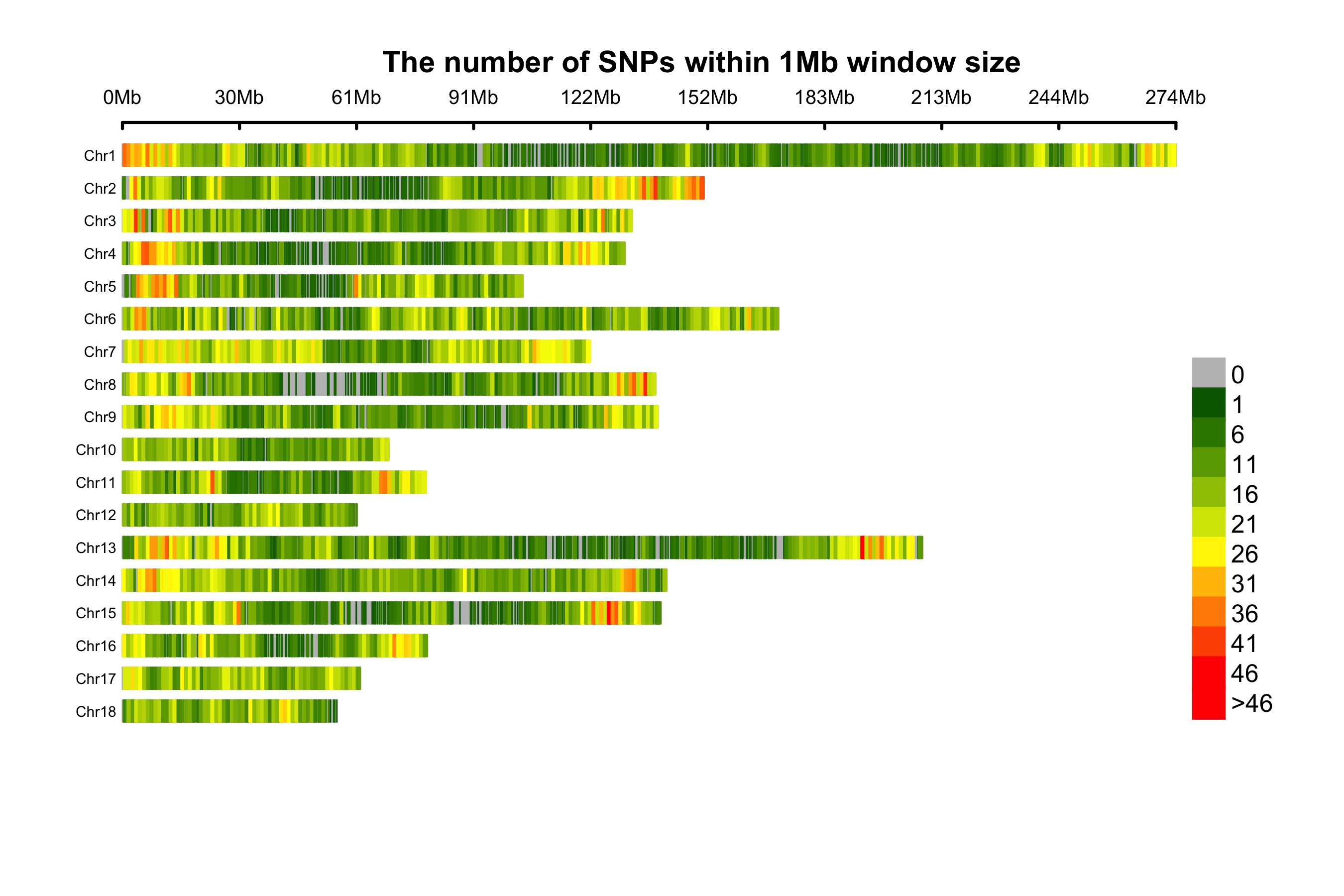


**Figure S1.** SNP density after quality control.

**Figure S2.** Principal component analysis of 508 animals.


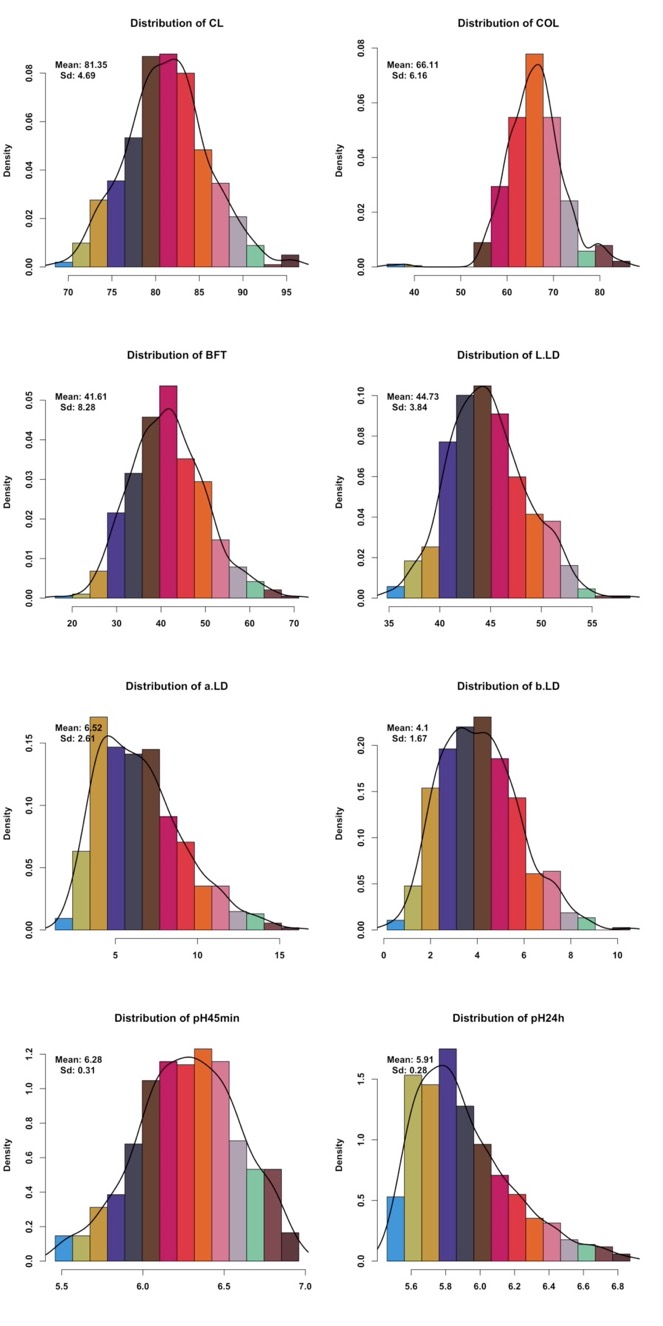


**Figure S3**. Distribution plot of eight traits in this study. Sd represents standard deviation.

**Figure S4**. Manhattan and Q-Q plots for four traits (b.LD, L.LD, pH_45min_, pH_24h_). The red dashed line is the genome-wide threshold (0.05/31,106). The -log_10_(*P*-value) of each SNP (y-axis) across the chromosomes (x-axis), along with the corresponding Q-Q plots. The λ represents genomic inflation factors. The Manhattan plot show the four traits have no significant SNP in this study.
