# Supplementary material for: Identifying Genetic Architecture of Carcass and Meat Quality Traits in a Ningxiang Indigenous Pig Population
Source: Genes (Basel). 2023 Jun 21;14(7):1308. doi: 10.3390/genes14071308 (PMC10378861; doi:10.3390/genes14071308)
Supplement: Supplementary file 1 [file genes-14-01308-s001.zip › genes-2419942 - Supplementary/genes-2419942 - Supplementary Table.docx]

**Table S1.** Abbreviation and measurement method description in this study.

| **Abbreviation** | **Description** | **Measurement** |
| --- | --- | --- |
| CW-Left (kg) | Left half carcass weight | The left half of the carcass weight of the pig shall be removed from the head, hoof, tail and viscera (to retain the suet and kidney) after bleeding and stretching. |
| LD | Longissimus dorsi | Collected the longissimus dorsi from the 6th to 12th ribs of the left carcass. |
| COL (cm) | Carcass oblique length | The oblique length from the front edge of pubic symphysis to the junction of the first rib and sternum measured by tape measure. |
| CL (cm) | Carcass length | Linear length from the front of pubic symphysis of the carcass to the front of the first cervical vertebra measured by tape measure. |
| BFT (mm) | Backfat thickness | The fat thickness of the carcass at the waist to sacrum junction measured by vernier caliper. |
| L.LD (%) | Longissimus dorsi (Lightness) | This trait was measured by colorimeter (SR-64). Repeat the measurement at 45 minutes after slaughter for three times to calculate the mean value. |
| a.LD (%) | Longissimus dorsi (Redness) | This trait was measured by colorimeter (SR-64). Repeat the measurement at 45 minutes after slaughter for three times to calculate the mean value. |
| b.LD (%) | Longissimus dorsi (Yellowness) | This trait was measured by colorimeter (SR-64). Repeat the measurement at 45 minutes after slaughter for three times to calculate the mean value. |
| pH_45min_ | pH after 45 minutes (Longissimus dorsi) | This trait was measured by Portable hand-held pH meter (HI-9025). Repeat the measurement at 45 minutes after slaughter for three times to calculate the mean value. |
| pH_24h_ | pH after 24 hours (Longissimus dorsi) | This trait was measured by Portable hand-held pH meter (HI-9025). Repeat the measurement at 24 hours after slaughter for three times to calculate the mean value. |

**Table S2.** Distribution of SNPs before, and after quality control and the average distance between adjacent SNPs on each chromosome.

| **CHR** | **NSNP^1^** | **NSNP^2^** | **Physical distance (bp)^3^** | **SNP/bp^4^** |
| --- | --- | --- | --- | --- |
| 1 | 4,753 | 3,327 | 274,018,613 | 82,386.83 |
| 2 | 3,212 | 2,204 | 151,322,033 | 68,689.08 |
| 3 | 2,840 | 1,861 | 132,538,400 | 71,257.2 |
| 4 | 2,938 | 1,691 | 130,756,997 | 77,371 |
| 5 | 2,239 | 1,459 | 103,541,477 | 71,016.1 |
| 6 | 3,338 | 2,439 | 170,514,723 | 69,940.41 |
| 7 | 2,697 | 2,219 | 121,206,582 | 54,646.79 |
| 8 | 2,857 | 1,644 | 138,698,852 | 84,418.05 |
| 9 | 3,081 | 1,869 | 139,185,075 | 74,510.21 |
| 10 | 1,453 | 946 | 69,170,958 | 73,196.78 |
| 11 | 1,810 | 1,115 | 78,978,619 | 70,896.43 |
| 12 | 1,276 | 877 | 60,934,130 | 69,559.51 |
| 13 | 3,573 | 2,557 | 207,996,524 | 81,375.79 |
| 14 | 3,214 | 2,164 | 141,617,056 | 65,472.52 |
| 15 | 2,834 | 1,754 | 140,055,764 | 79,894.90 |
| 16 | 1,774 | 1,124 | 79,253,250 | 70,572.80 |
| 17 | 1,376 | 1,027 | 61,598,438 | 60,037.46 |
| 18 | 1,235 | 829 | 55,566,607 | 67,109.43 |
| X | 2,192 | — | — | — |
| Y | 215 | — | — | — |
| Unknown | 1,790 | — | — | — |
| Total | 50,697 | 31,106 | 2,256,954,098 | 71,797.29 |

^1^ The original number of GGP50K chip.

^2^ Extracting criteria: MAF > 1%, HWE < 10^-6^, SNP call rate > 90%, autosomes. After quality control, we removed 537 SNPs due to HWE test, and 14,812 SNPs were removed due to MAF threshold.

^3^ Chromosome region length.

^4^ Average distance between SNPs.

**Table S3.** The genome-level significant and possible candidate genes for carcass and meat quality traits.

| **Trait** | **SNP** | **CHR** | **POS (bp)** | **Gene** |
| --- | --- | --- | --- | --- |
| CL | ALGA0040227 | 7 | 30176520 | *DAXX, SMIM40, KIFC1, PHF1, CUTA, SYNGAP1, ZBTB9, ITPR3, UQCC2, IP6K3, LEMD2, MIN,* ***GRM4****, HMGA1, SMIM29, NUDT3, RPS10, PACSIN1, SPDEF, ILRUN* |
|  | ALGA0040238 | 7 | 30197014 | *KIFC1, PHF1, CUTA, SYNGAP1, ZBTB9, ITPR3, UQCC2, IP6K3, LEMD2, MLN,* ***GRM4,*** *HMGA1, SMIM29,* *NUDT3, RPS10, PACSIN1, SPDEF, ILRUN* |
|  | INRA0024788 | 7 | 30317219 | *ITPR3, UQCC2, IP6K3, LEMD2, MLN, GRM4,* ***HMGA1****, SMIM29, NUDT3, RPS10, PACSIN1, SPDEF, ILRUN, SNRPC, BLTP3A, TAF11, ANKS1A* |
|  | ALGA0039917 | 7 | 26737102 | *TINAG,* ***MLIP****, LRRC1, KLHL31, GCLC* |
|  | ALGA0040777 | 7 | 36323988 | *LRFN2,* ***UNC5CL****, TSPO2, APOBEC2, OARD1, NFYA, TREML1, TREM2, TREM1, FOXP4* |
|  | ALGA0040243 | 7 | 30213771 | *PHF1, CUTA, SYNGAP1, ZBTB9, ITPR3, UQCC2, IP6K3, LEMD2, MLN,* ***GRM****4, HMGA1, SMIN29, NUDT3, RPS10, PACSIN1, SPDEF, ILRUN, SNRPC* |
|  | WU_10.2_7_48537179 | 7 | 41877149 | *CYP39A1, U6, SLC25A27, TDRD6, PLA2G7, ANKRD66, U6, ADGRF5,* ***ADGRF1****,* *U6,* *TNFRSF21, CD2AP* |
|  | ASGA0032589 | 7 | 31450019 | *ANKS1A, TCP11, U6, SCUBE3, ZNF76, DEF6, PPARD, FANCE, RPL10A, TEAD3, TULP1,* ***FKBP5****, ARMC12, CLPSL2, CLP5, LHFPL5, SRPK1, SLC26A8, MAPK13, BRPF3* |
|  | H3GA0020641 | 7 | 28521421 | ***PRIM2****, U6, RAB23, BAG2, ZNF451, BEND6, DST* |
|  | ALGA0039880 | 7 | 26501975 | *FAM83B,* ***TINAG****, MLIP, LRRC1* |
|  | ALGA0041948 | 7 | 50283279 | *CEMIP, MESD, U6, TLNRD1, CFAP161, IL16,* ***STARD5****, TMC3,* *MEX3B* |
|  | ALGA0040370 | 7 | 32328188 | *MAPK14, MAPK13, BRPF3, PNPLA1, BNIP5, ETV7, PXT1, KCTD20, STK38,* ***SRSF3****, CDKN1A, RAB44, CPNE5, PPIL1, C6orf89, PI16, MTCH1, FGD2, PIM1, TMEM217B, TMEM217* |
|  | M1GA0010006 | 7 | 31161760 | *ILRUN, SNRPC, BLTP3A, TAF11, ANKS1A, TCP11, U6, SCUBE3,* ***ZNF76****, DEF6, PPARD, FANCE, RPL10A, TEAD3, TULP1, FKBP5, ARMC12, CLPSL2, CLPS, LHFPL5, SRPK1* |
|  | WU_10.2_7_36255497 | 7 | 31181718 | *SNRPC, BLTP3A, TAF11, ANKS1A, TCP11, U6, SCUBE3,* ***ZNF76****, DEF6, PPARD, FANCE, RPL10A, TEAD3, TULP1, FKBP5, ARMC12, CLPSL2, CLPS, LHFPL5, SRPK1* |
|  | MARC0060950 | 7 | 46569153 | *IL17F, MCM3, PAQR8, EFHC1, TREM2,* ***TMEM14A****, GSTA4, CILK1, FBXO9, GCM1, U6, ELOVL5* |
| COL | ALGA0040227 | 7 | 30176520 | *DAXX, SMIM40, KIFC1, PHF1, CUTA, SYNGAP1, ZBTB9, ITPR3, UQCC2, IP6K3, LEMD2, MIN,* ***GRM4****, HMGA1, SMIM29, NUDT3, RPS10, PACSIN1, SPDEF, ILRUN* |
|  | ALGA0040238 | 7 | 30197014 | *KIFC1, PHF1, CUTA, SYNGAP1, ZBTB9, ITPR3, UQCC2, IP6K3, LEMD2, MLN,* ***GRM4,*** *HMGA1, SMIM29,* *NUDT3, RPS10, PACSIN1, SPDEF, ILRUN* |
|  | ALGA0039880 | 7 | 26501975 | *FAM83B,* ***TINAG****, MLIP, LRRC1* |
|  | H3GA0020641 | 7 | 28521421 | ***PRIM2****, U6, RAB23, BAG2, ZNF451, BEND6, DST* |
|  | ALGA0039917 | 7 | 26737102 | *TINAG,* ***MLIP****, LRRC1, KLHL31, GCLC* |
|  | INRA0024788 | 7 | 30317219 | *ITPR3, UQCC2, IP6K3, LEMD2, MLN, GRM4,* ***HMGA1****, SMIM29, NUDT3, RPS10, PACSIN1, SPDEF, ILRUN, SNRPC, BLTP3A, TAF11, ANKS1A* |
| BFT | WU_10.2_18_56654365 | 18 | 51759775 | *STK17A,* ***HECW1****, PSMA2, C7orf25* |
|  | WU_10.2_16_23509998 | 16 | 22361911 | *SLC1A3, U6,* ***NIPBL****, CPLANE1, NUP155, WDR70* |
|  | WU_10.2_8_138925750 | 8 | 129537879 | *MMRN1,* ***SNCA****, TIGD2, FAM13A* |
|  | ALGA0014052 | 2 | 82412427 | *HRH2, SFXN1, FCHO2, TMEM171,* ***TMEM174****, FOXD1, ANKRA2, UTP15, ARHGEF28* |
|  | ALGA0040227 | 7 | 30176520 | *DAXX, SMIM40, KIFC1, PHF1, CUTA, SYNGAP1, ZBTB9, ITPR3, UQCC2, IP6K3, LEMD2, MIN,* ***GRM4****, HMGA1, SMIM29, NUDT3, RPS10, PACSIN1, SPDEF, ILRUN* |
| a.LD | WU_10.2_16_23509998 | 16 | 22361911 | *SLC1A3, U6,* ***NIPBL****, CPLANE1, NUP155, WDR70* |
|  | WU_10.2_8_138925750 | 8 | 129537879 | *MMRN1,* ***SNCA****, TIGD2, FAM13A* |
|  | WU_10.2_18_56654365 | 18 | 51759775 | *STK17A,* ***HECW1****, PSMA2, C7orf25* |
|  | ALGA0014052 | 2 | 82412427 | *HRH2, SFXN1, FCHO2, TMEM171,* ***TMEM174****, FOXD1, ANKRA2, UTP15, ARHGEF28* |
|  | H3GA0000048 | 1 | 493510 | *PSMB1, DLL1,* ***ERMARD****, DYNLT2, PHF10, C1H6orf120, WDR27, THBS2* |

**Table S4.** Enrichment of KEGG pathway in Homo Sapiens dataset

| **Trait** | **KEGG Pathway** | **ID** | ***P-adj*** |
| --- | --- | --- | --- |
| a.LD | Proteasome | hsa03050 | 0.02 |
|  | RNA transport | hsa03013 | 0.04 |
| BFT | Glutamatergic synapse | hsa04724 | 0.03 |
| CL | Amyotrophic lateral sclerosis (ALS) | hsa05014 | 0.03 |
|  | Spliceosome | hsa03040 | 0.03 |
|  | Cellular senescence | hsa04218 | 0.04 |

**Table S5.** Top 10 traits with the highest enrichment QTLs number.

| **Trait** | **Related Trait Name** | **NQTLs** |
| --- | --- | --- |
| CL | Average daily gain | 13 |
|  | Average backfat thickness | 10 |
|  | Head Wight | 9 |
|  | Loin muscle area | 7 |
|  | Adipocyte diameter | 6 |
|  | Conductivity 45 minutes post-mortem | 6 |
|  | Backfat at last lumbar | 5 |
|  | Backfat at last rid | 5 |
|  | NADP-malate dehydrogenase activity | 4 |
|  | Carcass length | 3 |
| COL | Average daily gain | 14 |
|  | Average backfat thickness | 11 |
|  | Head Wight | 11 |
|  | Loin muscle area | 7 |
|  | Mean corpuscular volume | 7 |
|  | Adipocyte diameter | 6 |
|  | Conductivity 45 minutes post-mortem | 6 |
|  | Backfat at last lumbar | 5 |
|  | Backfat at last rid | 5 |
|  | Carcass Length | 4 |
| BFT | Average daily gain | 5 |
|  | Carcass weight(hot) | 3 |
|  | Average backfat thickness | 2 |
|  | Body weight | 2 |
|  | Fat-cuts percentage | 2 |
|  | Head weight | 2 |
|  | Intramuscular fat content | 2 |
|  | Loin muscle area | 2 |
|  | Loin muscle depth | 2 |
|  | Muscle moisture percentage | 2 |
| a.LD | Average daily gain | 4 |
|  | Average backfat thickness | 2 |
|  | Body weight | 2 |
|  | Ham weight | 2 |
|  | Intramuscular fat content | 2 |
|  | Leaf fat weight | 2 |
|  | 3 hours - 24 hours pH decline | 1 |
|  | Meat color a* | 1 |
|  | Meat color b* | 1 |
|  | Meat color L^*^ | 1 |
